# Association Study Indicates a Protective Role of Phosphatidylinositol-4-Phosphate-5-Kinase against Tardive Dyskinesia

**DOI:** 10.1093/ijnp/pyu098

**Published:** 2015-01-29

**Authors:** Olga Yu Fedorenko, Anton J. M. Loonen, Florian Lang, Valentina A. Toshchakova, Evgenia G. Boyarko, Arkadiy V. Semke, Nikolay A. Bokhan, Nikolay V. Govorin, Lyubomir I. Aftanas, Svetlana A. Ivanova

**Affiliations:** Mental Health Research Institute, SiberianBranch of RAMSc, Tomsk, Siberia, Russian Federation (Drs Fedorenko, Toshchakova, Boyarko, Semke, Bokhan, and Ivanova); National Research Tomsk Polytechnic University, Tomsk, Siberia, Russian Federation (Drs Fedorenko and Ivanova); Department of Pharmacy, University of Groningen, Groningen, The Netherlands (Dr Loonen); Mental Health Institute Westelijk Noord-Brabant, Halsteren, The Netherlands (Dr Loonen); Department of Physiology, University of Tuebingen, Tuebingen, Germany (Dr Lang); Chita State Medical Academy, Chita, Siberia, Russian Federation (Dr Govorin); National Research Tomsk State University, Tomsk, Siberia, Russian Federation (Dr Bokhan); Scientific Research Institute of Physiology and Basic Medicine, Siberian Branch of RAMSc, Novosibirsk, Siberia, Russian Federation (Dr Aftanas).

**Keywords:** PIP5K2A, schizophrenia, tardive dyskinesia, gene polymorphism, medium spiny neurons, neurotoxicity

## Abstract

**Background:**

Tardive dyskinesia is a disorder characterized by involuntary muscle movements that occur as a complication of long-term treatment with antipsychotic drugs. It has been suggested to be related to a malfunctioning of the indirect pathway of the motor part of the cortical-striatal-thalamic-cortical circuit, which may be caused by oxidative stress-induced neurotoxicity.

**Methods:**

The purpose of our study was to investigate the possible association between phosphatidylinositol-4-phosphate-5-kinase type IIa (PIP5K2A) function and tardive dyskinesia in 491 Caucasian patients with schizophrenia from 3 different psychiatric institutes in West Siberia. The Abnormal Involuntary Movement Scale was used to assess tardive dyskinesia. Individuals were genotyped for 3 single nucleotide polymorphisms in *PIP5K2A* gene: rs10828317, rs746203, and rs8341.

**Results:**

A significant association was established between the functional mutation N251S-polymorphism of the *PIP5K2A* gene (rs10828317) and tardive dyskinesia, while the other 2 examined nonfunctional single nucleotide polymorphisms were not related.

**Conclusions:**

We conclude from this association that PIP5K2A is possibly involved in a mechanism protecting against tardive dyskinesia-inducing neurotoxicity. This corresponds to our hypothesis that tardive dyskinesia is related to neurotoxicity at striatal indirect pathway medium-sized spiny neurons.

## Introduction

Dyskinesia is a collective name for a variety of involuntary hyperkinetic movements (Loonen and Van Praag, 2007). The movements are irregular, repetitive, and typically include motionless intervals. Dyskinesia may result from long-term treatment with antipsychotic drugs. This involuntary movement syndrome is termed tardive dyskinesia (TD) (Margolese et al., 2005; Kane, 2006). TD is a potentially disabling irreversible movement disorder, which has a prevalence of around 30% in patients chronically exposed to antipsychotics (Kane et al., 1988; Glazer, 2000). It can be subdivided into orofaciolingual (TDof) and limb-truncal (TDlt) dyskinesia (Al Hadithy et al., 2009, 2010).

**Figure 1.**
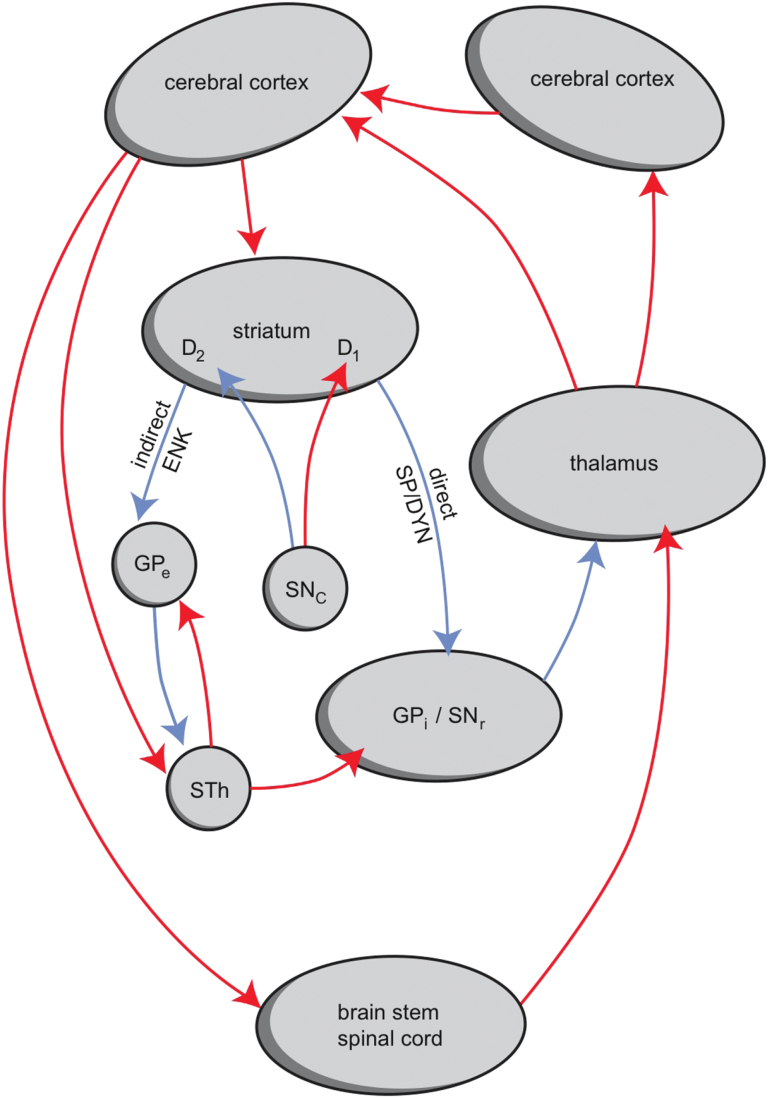
The cortical-striatal-thalamic-cortical circuits, including the indirect and direct pathways. Activation of the direct pathway causes hyperkinesia and activation of the indirect pathway causes hypokinesia. ENK, enkephalin; GPe, globus pallidus, external segment; GPi, globus pallidus, internal segment; SNc, substantia nigra, pars compacta; SNr, substantia nigra, pars reticulata; SP/DYN, substance P/dynorphin; STh, subthalamic nucleus; D1, D2, medium-sized spiny neurons (MSNs) with D1 or D2 receptors. Red, excitatory (glutamatergic, dopaminergic); blue, inhibitory (GABAergic, dopaminergic).

TD is classified as an extrapyramidal movement disorder and may be related to a malfunctioning of the indirect pathway of the motor part of the cortical-striatal-thalamic-cortical circuit (Figure 1) (Loonen and Ivanova, 2013). The indirect pathway starts with dopamine-D2 receptor expressing medium-sized spiny neurons (MSNs) in the striatum. Activation of this pathway results in inhibition of motor parts of the frontal cerebral cortex, and malfunctioning of this circuit would result in disinhibition and therefore hyperkinesia (Loonen and Ivanova, 2013).

Recently, our group identified an important link between 2 other hyperkinetic extrapyramidal movement disorders: Huntington’s disease (HD) and Levodopa-induced dyskinesia (LID). Patients suffering from LID are more often carriers of the same variants of the *GRIN2A* gene as are determining an earlier age of onset of dyskinesia in HD patients (Ivanova et al., 2012). The *GRIN2A* gene encodes for the NR2A subunit of the glutamatergic N-methyl-d-aspartate (NMDA) receptor (Paoletti and Neyton, 2007; Ivanova et al., 2012). In HD, symptoms are linked to NMDA receptor-induced excitotoxicity in indirect pathway MSNs (Estrada Sanchez et al., 2007; Fan and Raymond, 2007; Kumar et al., 2010). Our finding suggests that LID is related to a similar NMDA receptor-related malfunctioning of dopamine-D2 receptor carrying indirect pathway MSNs as HD. According to the neurotoxicity theory of TD, degeneration of indirect pathway MSNs in this disorder is related to neurotoxic effects of the free radicals produced by excessive metabolism of dopamine (Lohr et al., 2003). This theory suggests that antipsychotic drugs block dopamine D2 receptors and therefore trigger a compensatory release of excess dopamine. This excess requires increased metabolism of the spilled neurotransmitter. Increased dopamine metabolism releases high levels of hydrogen peroxide, which results in the production of free radicals, which then cause cell damage. Hence, excessive dopamine metabolism results in the production of more free radicals than the cell can handle. This hypothesis is consistent with the reported association between the incidence of TD and the presence of variants in the gene that encodes manganese superoxidedismutase, an enzyme that scavenges free radicals (Al Hadithy et al., 2010). A reduction in manganese superoxidedismutase activity would increase the likelihood of neurotoxic effects. It can be concluded that HD, LID, and TD are related to neurotoxic damage of indirect pathway MSNs and that every factor that increases neurotoxicity may also increase the likelihood of their becoming symptomatic.

Phosphatidylinositol 4-phosphate 5-kinase (PIP5K; EC 2.7.1.68) is a neuronal intracellular enzyme that produces phosphatidylinositol (4,5)-biphosphate, which is catalyzed by phospholipase C to the second messengers inositol (1,4,5) triphosphate and diacylglycerol (for review, see Van den Bout and Divecha, 2009). Three isoforms of this enzyme have been identified: PIP5Kα, PIP5Kβ, and PIP5Kγ. The PIP5Kα isoform is also known as phosphatidylinositol-4-phosphate-5-kinase type IIa (PIP5K2A) and localizes to the plasma membrane and the Golgi complex and in the nucleus. PIP5K2A is involved in many different processes, including signal transduction of G-protein-coupled receptors, cell survival by protection against apoptosis, and the genetic response to oxidative stress (Van den Bout and Divecha, 2009). The *PIP5K2A* gene has been shown to be associated with schizophrenia in several independent studies (Schwab et al., 2006; Bakker et al., 2007; He et al., 2007; Saggers-Gray et al., 2008; Fedorenko et al., 2013). This is possibly related to a similar direct vs indirect pathway MSN hyperactivity explaining positive psychotic symptoms in schizophrenia as well as dyskinesia in TD. Indeed, drug-naïve first-episode patients experience spontaneous dyskinesia more frequently than healthy controls (Tenback and Van Harten, 2011).

Although the exact regulatory functions of different types of PIP5Ks are far from evident, these enzymes can be expected to also play a role in augmenting or decreasing the excitability of corticostriatal glutamatergic synapses with MSNs during the induction of long-term potentiation and long-term depression (LTD), respectively. Long-term potentiation and LTD may play an important role in the mechanism of dyskinesia, as they regulate the readiness of corticostriatal synapses to excitatory (including excitotoxic) effects (Ivanova et al., 2012). In mice, for example, NMDA receptor-mediated compensatory LTD depends upon activation of PIP5Kγ661, which results in AMPA receptor endocytosis (Unoki et al., 2012).

In a heteromeric expression system, PIP5K2A has been disclosed to be a novel signaling element in the regulation of the neuronal KCNQ2/KCNQ3 and KCNQ3/KCNQ5 channels, EAAT3 glutamate transporter, and GluA1 function (Fedorenko et al., 2008, 2009; Seebohm et al., 2014). It has been shown that PIP5K2A regulation is disrupted in the schizophrenia-associated mutant *(N251S)-PIP5K2A* (rs10828317), which may contribute to the pathogenesis of schizophrenia through uncontrolled dopaminergic firing and deranged glutamate metabolism in the brain of schizophrenic patients carrying this mutation (Fedorenko et al., 2008, 2009; Seebohm et al., 2014).

We decided to study a possible association between a genetic variant of *PIP5K2A* encoding—according to in vitro observations (Fedorenko et al., 2008, 2009; Seebohm et al., 2014)—for a less active variant of this enzyme in comparison to 2 nonfunctional genetic variations and the prevalence of TD in a White Siberian patient population suffering from schizophrenia in order to establish a possible role for PIP5K in the pathophysiology of this disorder.

## Patients and Methods

### Patients

The work described in this article was carried out in accordance with the most recent version of the Code of Ethics of the World Medical Association (Declaration of Helsinki) for experiments involving humans and with the Uniform Requirements for manuscripts submitted to biomedical journals. After obtaining approval of the study protocol by the institutional bioethics committee, suitable participants were recruited from 3 psychiatric hospitals in the Tomsk, Kemerovo, and Chita areas in Siberia (Russia). All subjects gave informed consent after proper explanation of the study.

We included 491 subjects with a clinical diagnosis of schizophrenia the 10th revision of the International Statistical Classification of Diseases and Related Health Problems (ICD-10: F20; N=465; 94.7%) or schizotypal disorder (ICD-10: F21) and excluded subjects with non-Caucasian physical appearance (eg, Mongoloid, Buryats, Tyvans, or Khakassians) or those with organic or neurological disorders. Patients were assessed for the presence or absence of dyskinesia according to the abnormal involuntary movement scale (AIMS) (Loonen and Van Praag, 2007). The AIMS scores were transformed into a binary form (presence or absence of dyskinesia) with Schooler and Kane (1982) criteria. The presence of TDof and TDlt was established by a cutoff score of ≥2 (mild but definite) on any of the items 1 through 4 and 5 through 7 of AIMS, respectively. The sum of the first 4 items was used as a proxy for the severity of TDof, while the sum of items 5 thru 7 was used as a proxy for the severity of TDlt.

A blood sample was taken for DNA isolation and genotyping. The other inclusion criteria were no addictions, no organic disorders, and a high-quality DNA sample.

### Medication

On the day of TD assessment, a complete documentation of the medications utilized was compiled by the raters. For comparison, daily antipsychotic medication dosages were converted into chlorpromazine equivalents (Andreasen et al., 2010). Patients using clozapine who did not suffer from TD were excluded, as clozapine may suppress the symptoms of TD.

### Genotyping

DNA extraction was conducted according to standard protocols using phenol-chloroform extraction. Genotyping of *PIP5K2A* polymorphisms (rs10828317, rs746203, rs8341) was performed on an ABI StepOnePlus with a TaqMan Validateе SNP Genotyping Assay (Applied Biosystems).

### Statistics

The Hardy-Weinberg equilibrium of genotypic frequencies was tested by the chi-square test. Statistical analyses were performed using SPSS software, release 17, for Windows; *P*<.05 was considered as significant. To apply a correction for multiple testing, we used algorithm for False Discovery Rate control, described by Benjamini and Hochberg (1995).

The chi-square test and the Fisher’s exact test, if necessary, were used for between-group comparisons of genotypic or allelic frequencies. Between-group differences in continuous variables were evaluated using the Student’s *t* test or 1-way analysis of variance. Comparisons of AIMS-score in different groups were carried out with the Kruskal Wallis test. The relevant Bonferroni correction for multiple testing was applied.

Logistic regression analysis was performed to isolate the possible TD-related variables: age, sex, duration of illness, age at onset, and PIP5K2A polymorphism.

## Results

Table 1 shows the clinical and demographic characteristics of patients with and without TD. The genotype distribution of *PIP5K2A* (rs10828317, rs746203, rs8341) polymorphisms were in agreement with Hardy-Weinberg equilibrium in this patient group. No significant differences in genotype frequencies of the 2 nonfunctional polymorphisms rs746203 and rs8341 between the 2 groups of patients with and without TD were found (Table 2). However, a significant association was demonstrated to exist between TD and the functional rs10828317 mutation. After correction for multiple testing, the observed differences remained statistically significant (*P*=.018). CC carriers had a higher risk of TDof (OR=2.55, 95CI=1.56–4.14, *P*=.0006), TDlt (OR=1.85, 95CI=1.1–3.13, *P*=.04), and TDtot (OR=2.17, 95CI=1.34–3.51, *P*=.003). So, the frequency of CC-carriers is about twice as high in the group of schizophrenic patients with TD compared to the group without TD.

**Table 1.** The Clinical and Demographic Characteristics of Patients with and without TD

	With TH	Without TH	*p*
Gender (M/F)	83/48	221/139	X=.158; *P*=.691*
Age (y, mean±SD)	43.9±14.5	39.3±15.2	*P*=.003**
Age of onset (y, mean±SD)	24.95±8.8	24.98±8.9	*P*=.978**
Duration of disorder (y, mean±SD)	19±13.7	14.3±12.6	*P*=.000**

Abbreviation: TD, tardive dyskinesia.

*Chi-square test; ** *t* test.

**Table 2.** Distribution of rs10828317, rs8341, and rs746203 Genotypes and Alleles in Patients with and without TD

		Patients with TH	Patient without TH	Intergroup comparison, chi-square test
rs10828317 Genotype, N (%)	TT	44 (34.6)	144 (43.3)	X=10.306, *P*=.006
	CT	48 (37.8)	139 (41.9)	
	CC	35 (27.6)	49 (14.8)	
	T	0.56	0.64	
	C	0.46	0.36	
	HWE	X=7.33, *P* =.007	X=2.57, *P* =.11	
rs8341 Genotype	TT	14 (14.4)	41 (13.3)	X=0.615, *P*=.735
	CT	49 (50.5)	146 (47.2)	
	CC	34 (35.1)	122 (39.5)	
	T	0.4	0.37	
	C	0.6	0.63	
	HWE	X=0.2952, *P*=.6719	X=0.0669, *P*=.9016	
rs746203 Genotype	TT	33 (34.7)	118 (39.1)	X=0.8, *P* =.67
	CT	45 (47.4)	139 (46)	
	CC	17 (17.9)	45 (14.9)	
	T	0.58	0.62	
	C	0.42	0.38	
	HWE	X=0.0593, *P*=.8307	X=0.15, *P* =.7133	

Abbreviation: TD, tardive dyskinesia.

We also found an association between genotype and severity of TD. Patients who are CC carriers of rs10828317 had a significantly (*P*<.02 Mann-Whitney with Bonferroni correction) higher mean AIMS TDtot and TDof score in comparison to those with the CT or TT genotype (data not shown).

Analysis of covariance with age, sex, duration of disease, and chlorpromazine equivalent incorporated as covariates showed that TD is significantly (*P* < .005) associated with the *PIP5K2A* (rs10828317) polymorphism (details not shown).

Using the binary logistic regression method, we revealed an association between the CC-genotype of rs10828317 and TD (*P*=.005), whereas the input of age (*P*=.329), sex (*P*=.956), duration of disease (*P*=.139), chlorpromazine equivalent (*P*=.683), and age of onset of the disorder (*Р*=.608) were not statistically significant for our model.

## Discussion

**Figure 2.**
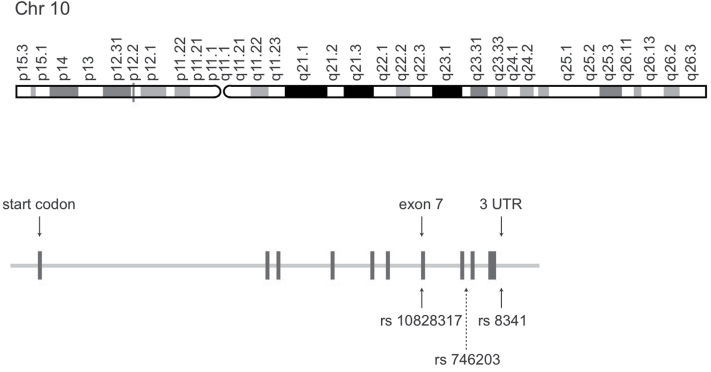
Representation of the single nucleotide polymorphism positions of 3 studied polymorphisms of the PIP5K2A gene (He et al., 2007).

In this study, we genotyped patients with and without TD with respect to 3 polymorphisms of the *PIP5K2A* gene. In Figure 2, the single nucleotide polymorphism positions are represented. Only one of them, rs10828317, is known to be a functional mutation. Replacement of T to C leads to a nonsynonymous amino-acid exchange (asparagine/serine) that causes an increased distance between 2 antiparallel helices from 3Å to 6Å and thereby interferes with the function of the enzyme (Fedorenko et al., 2008).

We decided to study the *PIP5K2A* gene, because this gene has repeatedly been shown to be associated with schizophrenia (Schwab et al., 2006; Bakker et al., 2007; He et al., 2007; Saggers-Gray et al., 2008), and the vulnerability to develop TD is related to the likelihood to develop positive symptoms of schizophrenia. In a previous study, we have confirmed this association in the presently studied Caucasian Siberian patients with schizophrenia (Fedorenko et al., 2013), but now we also demonstrated a relationship with the prevalence of TD. Koning et al. (2011a, 2011b) have described an association of TD with schizotypy in unaffected siblings of patients with nonaffective psychosis. Moreover, drug naïve first-episode patients sometimes show spontaneous dyskinesia (Tenback and Van Harten, 2011). Therefore, indirect evidence supports a possible role of genetic factors increasing the vulnerability to develop TD in patients with schizophrenia. Hereditary decreased activity of PIP5K might be one of them.

The present study did not address the mechanisms regulated by PIP5K2A and possibly contributing to the development of TD in carriers of the rs10828317 polymorphism. It is noteworthy, however, that PIP5K2A participates in the regulation of both glutamate receptor GluA1 (Seebohm et al., 2014) and glutamate carrier EAAT3 (Fedorenko et al., 2009). Thus, PIP5K2A may both increase glutamate sensitivity of neurons and terminate glutamate-induced excitation by accelerating clearance of glutamate from the synaptic cleft. It is tempting to speculate that deranged glutamate sensitivity or abundance may foster the development of TD, as it may increase the vulnerability of indirect pathway MSNs for oxidative stress-induced neurotoxicity (Loonen and Ivanova, 2013).

In conclusion, the present observations reveal an association of PIP5K2A gene variants with TD and thus suggest a clinical significance of this kinase in the control of movement and/or neuronal survival.

## Statement of Interest

None.
